# Deoxynivanelol and Fumonisin, Alone or in Combination, Induce Changes on Intestinal Junction Complexes and in E-Cadherin Expression 

**DOI:** 10.3390/toxins5122341

**Published:** 2013-11-28

**Authors:** Karina Basso, Fernando Gomes, Ana Paula Loureiro Bracarense

**Affiliations:** Laboratory of Animal Pathology, Veterinary Medecine Department, Universidade Estadual de Londrina, Rodovia Celso Garcia Cid, Km 380, PO Box 10.011, Londrina, Paraná 86057-970, Brazil; E-Mails: karinavet.basso@gmail.com (K.B.); fercg01@hotmail.com (F.G.)

**Keywords:** *Fusarium* spp., mycotoxins, gut, cell permeability, adherens junction

## Abstract

Fusariotoxins such as fumonisin B1 (FB1) and deoxynivalenol (DON) cause deleterious effects on the intestine of pigs. The aim of this study was to evaluate the effect of these mycotoxins, alone and in combination, on jejunal explants from piglets, using histological, immunohistochemical and ultrastructural assays. Five 24-day old pigs were used for sampling the explants. Forty-eight explants were sampled from each animal. Explants were incubated for 4 hours in culture medium and medium containing FB1 (100 µM), DON (10 µM) and both mycotoxins (100 µM FB1 plus 10 µM DON). Exposure to all treatments induced a significant decrease in the normal intestinal morphology and in the number of goblet cells, which were more severe in explants exposed to DON and both mycotoxins. A significant reduction in villus height occurred in groups treated with DON and with co-contamination. Expression of E-cadherin was significantly reduced in explants exposed to FB1 (40%), DON (93%) and FB1 plus DON (100%). The ultrastructural assay showed increased intercellular spaces and no junction complexes on enterocytes exposed to mycotoxins. The present data indicate that FB1 and DON induce changes in cell junction complexes that could contribute to increase paracellular permeability. The *ex vivo* model was adequate for assessing intestinal toxicity induced by exposure of isolated or associated concentrations of 100 µM of FB1 and 10 µM of DON.

## 1. Introduction

Mycotoxins are secondary metabolites produced by several fungal genera. They act as natural contaminants and are commonly found in grains and fresh foods of vegetable origin. It is estimated that 25% of the grains worldwide are contaminated with these substances [1]. A survey including 7049 samples collected in Europe, Asia and Americas revealed that about 64% and 59% of raw materials and finished feed samples contained fumonisins (FB) and deoxynivalenol (DON), respectively. The mean levels were 1 mg/kg (maximum of 49 mg/kg) for DON and 2 mg/kg (maximum of 77 mg/kg) for FB [2]. 

The fumonisin B1 (FB1) corresponds to 70% of fumonisins and is produced by *Fusarium verticillioides* [3]. The toxicity of FB1 has been proved in several animal species, resulting in different effects such as acute pulmonary edema in pigs, leukoencephalomalacia in horses, liver cancer in rats and esophageal cancer in humans [4]. Data on the mechanisms of action of FB1 on the digestive system are scarce, and routes of action on the intestinal epithelium are poorly understood. Exposure to FB1 induces a reduction in cell number due to decrease in cell proliferation associated with increased apoptotic index, as well as a decrease in transepithelial electrical resistance, indicating changes in intestinal integrity [5]. *In vivo* studies in piglets demonstrated that acute and chronic ingestion of feed contaminated with fumonisin led to a significant increase in hepatic [6] and intestinal lesions such as atrophy and fusion of villi, and decreased E-cadherin expression [7]. Piglets exposed to this mycotoxin showed higher bacterial translocation to various organs [8], favoring the proliferation of opportunistic bacteria in the gut [9].

The fusariotoxin deoxynivalenol (DON) frequently contaminate corn and wheat, being a risk to human and animal health [10,11,12,13]. Exposure of intestinal explants to DON causes morphological changes in a dose-dependent manner, such as flattening of enterocytes; villi atrophy and increased apoptotic index [14]. One effect of this mycotoxin on the intestine is the reduction in the expression of proteins cell junctions as claudin-4 [15,16], E-cadherin and occludin [7], resulting in changes in paracellular and transcellular permeability, favoring penetration of pathogens [13,15,16,17]. 

The ultrastructural evaluation may help in understanding the pathophysiology of injury; however studies on the effects of mycotoxins on intestinal ultrastructure are scarce [18,19]. There is no data in the literature on ultrastructural changes induced by exposure of the bowel to fumonisins and deoxynivalenol. Health regulations only consider the effects of mono-contamination, but multi-contamination is a phenomenon often observed in natural contamination of feed [6]. The available data indicate that simultaneous intake of FB1 and DON induces an additive immunosuppressive effect as compared with exposure to a single toxin [6,7]. The need for more research into additive, synergistic or antagonistic effects in multi-contamination is necessary; however, *in vivo* studies are costly and involve bioethical issues. Thus, the use of alternative models which mimic the organic systems of interest is extremely interesting. The efficacy of *ex vivo* model for assessing the effects of exposure to DON on the intestine has been proven in previous studies [14,20]. The model is also appropriate to examine the expression of protein junctions of enterocytes [21]. Considering the need to broaden knowledge about the results of interactions between multiple mycotoxins and the limited available data, the aim of this study was to assess the effects of exposure to FB1 and DON, alone and in combination, with emphasis on E-cadherin expression and ultrastructural changes, using the *ex vivo* model of intestinal explant. 

## 2. Results

### 2.1. Histological Analysis

**Figure 1:**
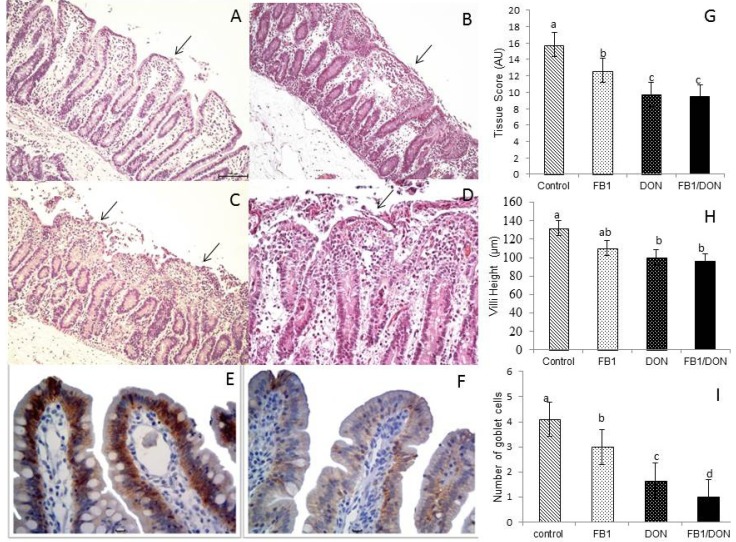
Effects of individual and combined exposition of jejunal explants to fumonisin and deoxynivalenol on histology. Explants were exposed to culture medium (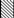) or culture medium with fumonisin B1 (FB1) (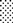), deoxynivalenol (DON) (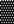) or FB1 + DON (■). (**A**) Control explants. Edema of the lamina propria and mild villi atrophy (arrow); (**B**) FB1-exposed explant. Moderate fusion (arrow) and villous atrophy; (**C**) DON-exposed explant. Severe loss of apical enterocytes, fusion and atrophy (arrow); (**D**) FB1 + DON-exposed explant. Lysis of intestinal epithelium, villi atrophy, fusion (arrow) and cell debris. HE. Bar 100 µm; (**E**) Control explant showing a strong and homogeneous E-cadherin expression. Bar 20 µm; (**F**) DON-exposed explant showing reduced expression of E-cadherin. Bar 20 µm; (**G**) Tissue scores of pig intestinal explants exposed to FB1, DON and both mycotoxins; (**H**) Villi height in pig intestinal explants treated with FB1, DON and FB1 + DON; (I) Number of goblet cells per villus of pig intestinal explants treated with FB1, DON and FB1 + DON. Values are means with their standard deviation of the mean represented by vertical bars (*n* 5 animals). Mean values with unlike letters were significantly different (*p* ≤ 0.05). AU = Arbitrary Units.

The main histological changes observed in the control group were edema of lamina propria, mild cell degeneration and villi atrophy (Figure 1A). In explants exposed to FB1, flattening and focal loss of apical enterocytes, moderate fusion and villi atrophy were observed (Figure 1B). In the group treated with DON, the changes are similar to the FB1 group; however the intensity of the lesions was more severe and a reduction in villi number was also verified (Figure 1C). Explants exposed to both mycotoxins showed severe changes characterized by reduction in villi number, villi fusion and atrophy, besides lysis of enterocytes (Figure 1D). A significant decrease in histological score was observed in all explants treated with mycotoxins when compared to control group (Figure 1G). The reduction was 18.8%, 37% and 37.5% for exposure to FB1, DON and FB1 plus DON, respectively. The morphometric analysis showed a significant decrease in villi height in the explants treated with DON and FB1 plus DON (*p* ≤ 0.05) compared to the control group (Figure 1H).

The explants exposed to all treatments showed a significant change in the number of goblet cells (*p* ≤ 0.05) in comparison to control explants. The reduction was more pronounced in the groups treated with FB1 plus DON (98%) and DON (63.4%) (Figure 1I). 

### 2.2. Expression of E-Cadherin

The immunohistochemical analysis was performed to evaluate the expression of the adherens junction protein, E-cadherin. We observed a significant decrease in expression of E-cadherin in the group treated with FB1 (40%) and DON (93%) compared to control (Figure 1E,F). The group treated with both mycotoxins showed no immunostaining for E-cadherin. 

### 2.3. Ultrastructural Analysis

**Figure 2:**
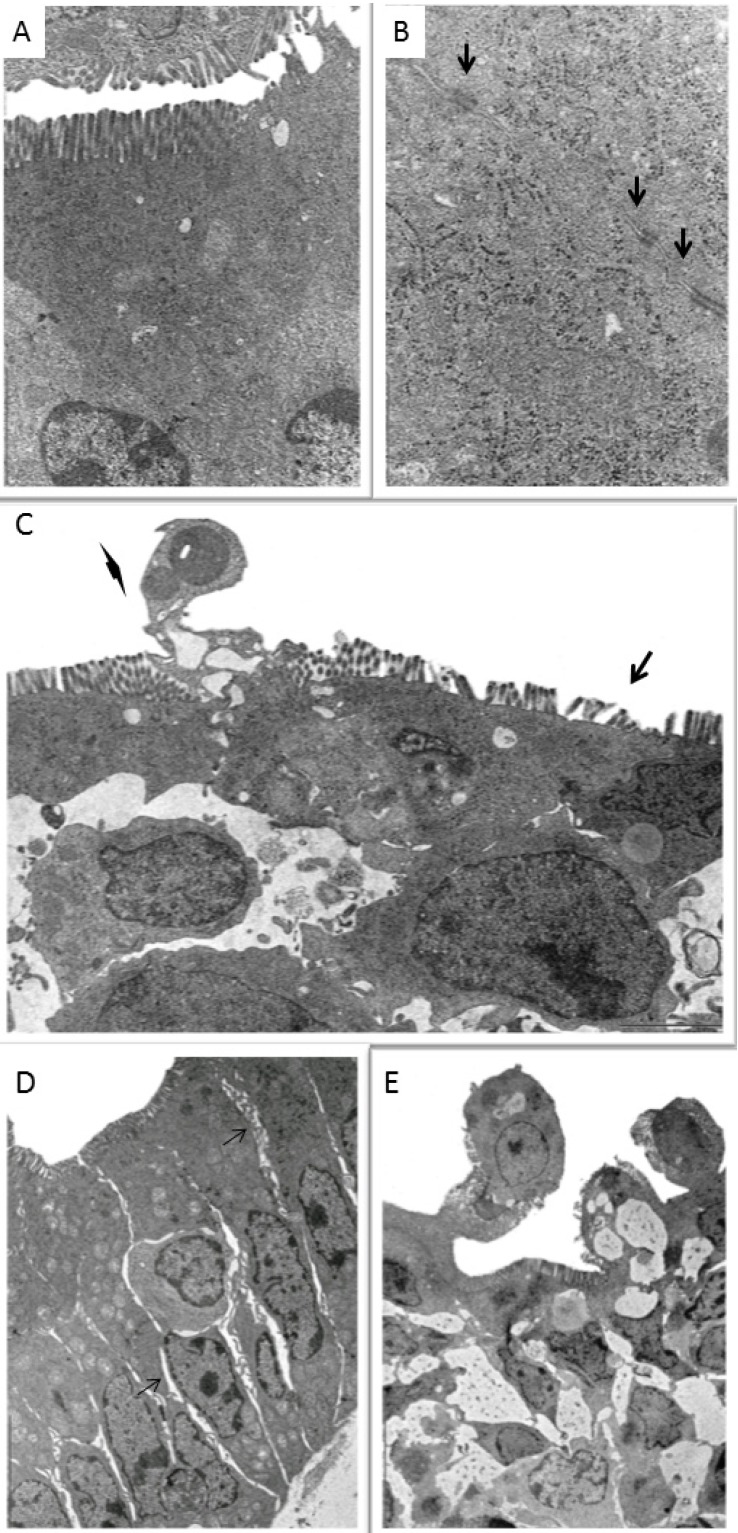
Effects of individual and combined exposition of jejunal explants to fumonisin and deoxynivalenol on ultrastructure. (**A**) Control explant. Enterocytes with normal morphology of microvilli and cytoplasm. Bar 500 nm; (**B**) Control explant. Enterocytes with junction complexes (arrow) and glycogen granules scattered in the cytoplasm. Bar 2 nm; (**C**) FB1-exposed explant. Focal loss of apical enterocytes (arrow head) and loss of microvilli (arrow). Bar 2 nm; (**D**) DON-exposed explant. Increased intercellular space (arrow) and loss of junction complexes. Bar 5 nm; (**E**) DON-exposed explant. Vacuoles within cytoplasm, membrane blebs and loss of apical enterocytes. Bar 10 nm.

Cell viability and the integrity of the complex of enterocyte junctions in explants exposed to fusariotoxins were evaluated by ultrastructural analysis. Control explants showed a continuous monolayer of enterocytes lining the gut. The luminal membrane of enterocytes presented preserved microvilli, while the apical membrane between adjacent intestinal cells formed intercellular junctions and desmosomes (Figure 2A,B). The cytoplasm was homogeneous with no changes in organelles. The nuclei contained a low concentration of heterochromatin and large, distinct nucleoli. Goblet cells were observed between enterocytes, showing secretory granules throughout the length of the villi. On the other hand, explants exposed to mycotoxins presented increased intercellular spaces, decrease in the size and number of microvilli (Figure 2C,D). Junction complexes were not visualized between the enterocytes. The nucleus showed marginated heterochromatin and occasionally apoptotic corpuscles. The presence of cytoplasmic projections, vacuoles within the cytoplasm, cellular debris and desquamated cells in the lumen were observed mainly in the explants incubated with DON (Figure 2E). The group exposed to both mycotoxins showed so severe changes that impaired ultrastructural evaluation. In this group we observed only cellular debris with no preserved enterocytes.

## 3. Discussion

The integrity of the gut is dependent on the maintenance of various factors, including enterocyte and mucus layer integrity, as well as preservation and functionality of epithelial junctions cells [21]. There is increasing evidence that the intestinal epithelium is repeatedly exposed to mycotoxins, and at a higher concentration than other tissues [22]. The ingestion of fusariotoxins may induce changes on intestinal morphology and local immunity [7] affecting the barrier function of the gut. The effects of mycotoxins on gastrointestinal tract can diverge because the bioavailability of these fungal compounds is very diverse and differs between animal species [22]. In this study, we used an *ex vivo* model to demonstrate that fusariotoxins alone or in combination induced significant morphological and ultrastructural changes in jejunal explants from pigs. 

The gut, as the first tissue to have contact with food contaminants, is considered a target organ for the action of mycotoxins [23]. In this experiment, we verified that exposition to DON, FB1 or both mycotoxins induced a significant decrease in intestinal score. Changes were more severe in explants incubated with DON and DON plus FB1. The main histological changes were lysis of enterocytes and villi atrophy and fusion. These histological findings are similar to those described in pigs chronically fed with FB1 and DON [7,24,25,26], demonstrating that the *ex vivo* model is suitable for assessing the effects of toxic substances on the intestine. It is interesting to observe that in both *ex vivo* and *in vivo* models a good correlation was reported for histological changes and phosphorylation of mitogen-activated kinases on intestine, even with differences in time of mycotoxin exposure (hours and days, respectively) [20,26]. In explants the morphological changes induced by fusariotoxins are more severe. Probably, the severity of the lesions on the explants is associated with a direct exposure to the toxin, whereas in the *in vivo* model the bioavailability of mycotoxins differs. In addition, the explants were submitted to a relative degree of hypoxia, a fact that can contribute to the intensity of histological changes.

It is worth noting that even with a short period of incubation with mycotoxins both toxins were able to cause significant injury on intestinal tissue. Deoxynivalenol induces a ribotoxic stress, compromising protein synthesis and triggering apoptosis [27], which may explain the microscopic changes observed in this study. Fumonisin leads to accumulation of sphinganine and sphingosine products that are cytotoxic, pro-apoptotic and cell growth inhibitors [28].

Changes in villi height imply in reduced absorption of nutrients [22]. In this study we verified a significant decrease in villi height in explants exposed to DON (24%) and DON plus FB1 (27%). Similar results were reported in pigs fed chronically diets contaminated with DON [7]. The reduction in villi height induced by DON is related to an increase in enterocyte apoptosis (data not shown). DON induces phosphorylation of mitogen-activated protein kinases (MAPKs) resulting in cell apoptosis [26]. In addition, DON is also able to reduce cell proliferation *in vitro* [27]. 

Regarding the effects of FB1 on the intestine, the results are conflicting. Doses of 30 mg/kg and 2.8 mol/Kg caused villus atrophy and fusion [29]; however, ingestion of a single dose of 83 mg/kg FB1 [3] or of 6 mg/kg chronically [7] induced no changes in villi height. In this study, explants exposed to FB1 showed no significant changes in villi height (reduction of 16.4%) compared to control explants. 

Goblet cell density reflects mucus production, an important protective intestinal barrier that prevents adhesion of pathogens to enterocytes [30]. In this experiment, explants exposed to all treatments showed a significant change in the number of goblet cells. We observed a reduction that was more severe in DON and FB1 plus DON treatments. Chronic ingestion of FB1 induced no changes in globet cell density in piglets (6 mg/Kg FB) [7], whereas in poultry (300 mg/kg) a significant increase was observed [31]. These differences are probably related to the doses of FB1 used. The explants were exposed to 100 µM, which is equivalent to a dose of 144 mg/kg of FB1. Also, pigs are much more susceptible to fumonisins than poultry [22]. Reduction in the number of goblet cells after chronic exposure to DON intestinal has already been reported in *in vivo* studies [7]. In a previous study, jejunal explants exposed to DON and FB1 exhibited a significant increase in caspase-3 immunostaining, indicating cell apoptosis (unpublished data). We hypothesize that the changes in goblet cell staining are also related to increased apoptosis induced by mycotoxins exposition. On the other hand, changes in goblet cells staining could be associated with two other mechanisms, expulsion of mucin granules and necrosis. DON induces a ribotoxic stress [27] that results in decreased synthesis of proteins such as mucin, while FB1 induces apoptosis by activation of the caspase-3 pathway [32]. The explants exposed to FB1 plus DON showed a drastic reduction in the number of goblet cells suggesting an additive effect of these mycotoxins on globet cells. 

A decrease in the expression of proteins cell junction and the induction of apoptosis are some of the factors associated with the deleterious effects of mycotoxins on the intestine [15]. Junction cell proteins as occludin, claudin and E-caderin have a fundamental role in maintaining epithelial architecture [33]. In this experiment we observed a decrease in E-cadherin expression, mainly in explants exposed to DON and to both mycotoxins. It is known that pigs chronically fed diets contaminated with DON, FB1 or FB1 plus DON present a significant decrease in E-cadherin and occludin expression [7]. In pigs’ intestinal cells exposed to DON, the changes in claudin-4 expression, a junction protein, were related to phosphorylation of ERK 1/2, a mitogen activated protein kinase [16]. The molecular mechanisms underlying FB1 exposition and changes in junction proteins remain to be investigated. The reduction in the expression of E-cadherin reflects the ability of mycotoxins to alter epithelial integrity; however future studies evaluating other proteins that comprise the junctional complex can establish the types of changes on the epithelium and the courses of action of mycotoxins. A decreased expression of E-cadherin is a factor that contributes to increased paracellular permeability and facilitates the invasion of pathogens. 

One of the aims of this study was to characterize the ultrastructural changes on the intestine induced by DON and FB1 with emphasis on cell junctions. To the best of our knowledge, this is the first study reporting the effects of DON and FB1 on intestinal ultrastructure. In this study we observed an increase in intercellular spaces and disappearance of junction structures induced by both mycotoxins. Increase in paracellular permeability has been reported in Caco-2 cells exposed to DON through inhibition of protein synthesis [34]. In addition, IPEC cells incubated with FB1 showed a decrease in transepithelial electrical resistance [5]. Our results indicate that DON and FB1 induce severe changes in junction complexes and cell structure increasing intestinal permeability and decreasing intestinal absorption. The ultrastructural findings observed on explants exposed to mycotoxins also strengthen those observed on immunohistochemical evaluation of E-cadherin expression reinforcing the changes on junction structures.

## 4. Material and Methods

### 4.1. Animals

Five piglets (Landrace × Large White × Duroc) of 24 days old (6.3 kg, ± 0.8) were used to collect the explants. The animals were weaned at 21 days of age, and then subjected to a standard diet after weaning in separate bays. At 24 days the piglets were euthanized with an intravenous injection of sodium pentobarbital (40 mg/kg of body weight). The institutional Ethics Committee for Animal Experimentation approved all animal procedures.

### 4.2. Culture of Explants and Exposure to FB1 and DON

The jejunum was chosen because in prior studies this region was shown to be more sensitive to the effects of mycotoxins [16]. Fragments of 5 cm of medial jejunum were sampled immediately after euthanasia, and washed with buffered saline (PBS) and opened longitudinally. The explants were collected with the aid of a biopsy punch (8 mm) and placed in six-well plates (EasyPath, São Paulo, Brazil), filled with 3 ml of agar and containing DMEM (GIBCO, NY, USA) plus fetal bovine serum (10%), glutamine (0.2 mL/L), gentamicin (0.5 mg/mL) and penicillin/streptomycin (10 mL/L). 

The explants were deposited with the mucosa facing upwards (3 explants/well) and incubated at 37 °C in the presence of FB1 (F1147, Sigma-Aldrich, São Paulo Brazil) (100 µM) or DON (D0156, Sigma-Aldrich, São Paulo, Brazil) (10 µM) alone and FB1 (100 µM) plus DON (10 µM) for four hours. The doses used in this experiment are based in previous studies *in vitro* [35] and *ex vivo* [20] that have shown toxic effects with these concentrations. The dose of FB1 (100 µM) corresponds to an intake of 144 mg/kg of feed, while the dose of DON (10 µM) corresponds to an ingestion of 3 mg/kg of contaminated feed. From each animal 48 explants were sampled and submitted to the different treatments (24 explants/animal were used for immunohistological analysis and the other 24 for ultrastructural analysis). A total of 240 explants were evaluated by immunohistological and ultrastructural methods. For each treatment three explants immersed in culture medium without mycotoxins (control group) were incubated. 

### 4.3. Histological and Morphometric Analysis

After the incubation period, explants were fixed in 10% buffered formalin solution, dehydrated in increasing alcohols and embedded in paraffin. Sections of 5μm were stained with hematoxylin and eosin (HE) for histological analysis. The histological changes were evaluated using an adapted tissue score [15] in which the intensity and severity of lesions were considered. The maximum score (22 points) indicates the overall integrity of the intestine. The criteria used to determine the score were morphology and number of villi, morphology of enterocytes and microvilli, presence of cellular debris, interstitial edema, lymphatic dilation and cellular necrosis. The lesion score was calculated by taking into account the degree of severity (severity factor) and the extent of each lesion (according to intensity or observed frequency, scored from 0 to 3). For each lesion, the score of the extent was multiplied by the severity factor.

To evaluate the density of goblet cells, sections were stained with alcian-blue. The number of goblet cells was counted in 10 randomly villi, using a 10 × magnification. Goblet cells present in the crypts were not counted. For morphometric evaluation the heights of 10 intestinal villi were measured randomly with the aid of the Motic Image Plus 2.0 software using a 10 × magnification.

### 4.4. Immunohistochemical Analysis

The expression of E-cadherin was evaluated using specific monoclonal antibody (clone 4A2C7, Zymed, Carlsbad, CA, USA) and the proportion of the intestinal section expressing E-cadherin was estimated. Each sample was assessed as showing either normal or reduced staining. Normal staining was considered when homogeneous and strong basolateral membrane staining of the enterocytes was detected. Heterogeneous and weak staining was considered to indicate reduced expression. The results are reported as the percentage of animals showing strong/homogeneous immunoreactivity to E-cadherin.

Tissue sections were deparaffinised with xylene and dehydrated through a graded ethanol series. Heat-mediated retrieval was done by heating the sections immersed in citrate buffer (pH 6.0) in a microwave oven (750 W) for 15 min. Endogenous peroxidase activity was blocked by incubation in methanol-hydrogen peroxide solution. The sections were incubated overnight at 4 °C with the primary antibody (diluted 1:50). The secondary antibody (Nichirei Biosciences, Tokyo, Japan) was applied followed by the addition of a chromogen (3,3′-diaminobenzidine). Finally, tissue sections were counterstained with hematoxylin and mounted on coverslips using a synthetic resin. All reactions were accompanied with negative and positive controls of the reaction according to the manufacturer. 

### 4.5. Ultrastructural Analysis

The explants exposed to the different treatments were submitted to transmission electron microscopy. After the incubation period samples were fixed in Karnovsky modified solution and post-fixed in 1% osmium tetroxide. After complete sequential dehydration, the samples were embedded in epoxy resin and maintained for 3 days at 60 °C for polymerization. Ultrathin sections were stained with uranyl acetate and lead citrate and analyzed by TEM (model FEI Tecnai 12).

### 4.6. Statistical Analysis

The data used for statistical analysis were represented as means with their standard deviation. The experimental design used in the present study was entirely randomized with six replicates (each explant representing one replicate). Oneway analysis of variance (ANOVA) followed by a multiple comparison procedure (Tukey test) was used for statistical analysis. Data of the expression of E-cadherin were subjected to Fisher's exact test. The lack of normality or homogeneity of goblet cell number leads to the use of the non-parametric Kruskal-Wallis and Dunn tests. Differences were considered statistically significant at *p* ≤ 0.05. Statistical analyses were performed with BioStat 5.0 (Belém, Pará, Brazil) software package. Ultrastructure parameters were subjected to a descriptive analysis.

## 5. Conclusions

In conclusion, results of the present study indicate that 10 µM of DON and 100 µM of FB1 alone or in combination induce significant damage on intestinal tissue. An association between exposition to these mycotoxins and reduced villi height, globet cell density, E-cadherin expression and junction complexes was also demonstrated. Taken together, these results support that DON and FB1 could increase enterocyte paracellular permeability and promote major changes on intestinal barrier function.

## References

[1] Cast I. (2003). Mycotoxins—Risks in Plant, Animal and Human Systems.

[2] Rodrigues I., Naehrer K. (2012). A three-year survey on the worldwide occurrence of mycotoxins in feedstuffs and feed. Toxins.

[3] Dilkin P., Direito G., Simas M.M.S., Mallmann C.A., Corrêa B. (2010). Toxicokinetics and toxicological effects of single oral dose of fumonisin B1 containing *Fusarium verticillioides* culture material in weaned piglets. Chemico-Biol. Interact..

[4] Minami L., Meirelles P.G., Hirooka E.Y., Ono E.Y.S. (2004). Fumonisinas: Efeitos toxicológicos, mecanismo de ação e biomarcadores para avaliação da exposição. Semina.

[5] Bouhet S., Hourcade E., Loiseau N., Fikry A., Martinez S., Roselli M., Galtier P., Mengheri E., Oswald I.P. (2004). The mycotoxin fumonisin B1 alters the proliferation and the barrier function of porcine intestinal epithelial cells. Toxicol. Sci..

[6] Grenier B., Bracarense A.P.F.R.L., Lucioli J., Pacheco G.D., Cossalter A.M., Moll W.D., Schatzmayr G., Oswald I.P. (2011). Individual and combined effects of subclinical doses of deoxynivalenol and fumonisin in piglets. Mol. Nutr. Food Res..

[7] Bracarense A.P., Lucioli J., Grenier B., Pacheco G.D., Moll W.D., Schatzma Y.R.G., Oswald I.P. (2012). Chronic ingestion of deoxynivalenol and fumonisin, alone or in interaction, induces morphological and immunological changes in the intestine of piglets. Br. J. Nutr..

[8] Oswald I.P., Desautels C., Lafftte J., Fournout S., Peress Y., Odin M., Bars L.E., Bars J., Fairbrother J.M. (2003). Mycotoxin fumonisin B1 increases intestinal colonization by pathogenic Escherichia coli in pigs. Appl. Environ. Microbiol..

[9] Maresca M., Yahi N., Younès-Sakr L., Boyron M., Caporiccio B., Fantini J. (2008). Both direct and indirect effects account for the pro-inflammatory activity of enteropathogenic mycotoxins on the human intestinal epithelium: Stimulation of interleukin-8 secretion, potentiation of interleukin-1 β effect and increase in the transepithelial passage of commensal bacteria. Toxicol. Appl. Pharmacol..

[10] Pestka J.J. (2010). Deoxynivalenol: Mechanisms of action, human exposure, and toxicological relevance. Arch. Toxicol..

[11] Döll S., Dänicke S. (2011). The fusarium toxins deoxynivalenol (DON) and zearalenone (ZON) in animal feeding. Prev. Vet. Med..

[12] Maresca M. (2013). From the gut to the brain: Journey and pathophysiological effects of the food-associated trichothecene mycotoxin deoxynivalenol. Toxins.

[13] Maresca M., Fantini J. (2010). Some food-associated mycotoxins as potential risk factors in humanspredisposed to chronic intestinal inflammatory diseases. Toxicon.

[14] Kolf-clauw M., Castellote J., Joly B., Bourges-Abella N., Raymond-Letron I., Pinton P., Oswald I.P. (2009). Development of a pig jejunal explant culture for studying the gastrointestinal toxicity of the mycotoxin deoxynivalenol: Histopathological analysis. Toxicol. Vitro.

[15] Pinton P., Nougayrède J.P., Del Rio J.C. (2009). The food contaminant deoxynivalenol, decreases intestinal barrier permeability and reduces claudin expression. Toxicol. Appl. Pharmacol..

[16] Pinton P., Braicu C., Nougayrede J., Lafitte J., Taranu I., Oswald I.P. (2010). Deoxynivalenol impairs porcine intestinal barrier function and decreases the protein expression of claudin-4 through a mitogen-activated protein kinase-dependent mechanism. J. Nutr..

[17] Vandenbroucke V., Croubels S., Martel A., Verbrugghe E., Goossens J., Deun K.V., Boyen F., Thompson A., Shearer N., de Backer P. (2011). The mycotoxin deoxynivalenol potentiates intestinal inflammation by *Salmonella* Typhimurium in porcine ileal loops. PloS One.

[18] Witloc D.R., Waytt R.D., Ruff M.D. (1977). Morphological changes in the avian intestine induced by citrinin and lack of effect of aflatoxin and T-2 toxin as seen with scanning electron microscopy. Toxicon.

[19] Obremski K., Gajecka M., Zielonka L., Jakimiuk E., Gajecki M. (2005). Morphology and ultrastructure of small intestine mucosa in gilts with zearalenone mycotoxicosis. Pol. J. Vet. Sci..

[20] Lucioli J., Pinton P., Callu P., Laffitte J., Grosjean F., Kolf-Clauw M., Oswald I.P., Bracarense A.P.F.R.L. (2013). The food contaminant deoxynivalenol activates the mitogen activated protein kinases in the intestine: Interest of *ex vivo* models as an alternative to *in vivo* experiments. Toxicon.

[21] Randall K., Turton J., Foster J.R. (2011). Explant culture of gastrointestinal tissue: A review of methods and applications. Cell. Biol. Toxicol..

[22] Grenier B., Applegate T.J. (2013). Modulation of intestinal functions following mycotoxin ingestion: Meta-analysis of published experiments in animals. Toxins.

[23] Bouhet S., Oswald I.P. (2007). The intestine as a possible target for fumonisin toxicity. Mol. Nutr. Food. Res..

[24] Lessard M., Boudry G., Sève B., Oswald I.P., Lallès J.P. (2009). Intestinal physiology and peptidase activity in male pigs are modulated by consumption of corn culture extracts containing fumonisins. J. Nutr..

[25] Zielonka L., Wiśniewska M., Obremski K., Gajęcki M. (2009). Influence of low doses of deoxynivalenol on histopathology of selected organs of pigs. Pol. J. Vet. Sci..

[26] Pinton P., Tsybulskyy D., Lucioli J., Laffitte J., Callu P., Lyazhri F., Grosjean F., Bracarense A.P.F.R.L., Kolf-Clauw M., Oswald I.P. (2012). Toxicity of deoxynivalenol and its acetylated derivatives on the intestine: Differential effects on morphology, barrier function, tight junctions proteins and mitogen-activated protein kinases. Toxicol. Sci..

[27] Bae H.K., Pestka J.J. (2008). Deoxynivalenol induces p38 interaction with the ribosome in monocytes and macrophages. Toxicol. Sci..

[28] Voss K.A., Smith G.M., Hascheck W.M. (2007). Fumonisins: Toxicokinetics, mechanism of action and toxicity. Animal Feed Sci. Technol..

[29] Dilkin P., Hassegawa R., Reis T.A., Mallmann C.A., Corrêa B. (2004). Intoxicação experimental de suínos por fumonisinas. Cienc. Rural..

[30] McGuckin M.A., Lindén S.K., Sutton P., Florin T.H. (2011). Mucin dynamics and enteric pathogens. Nat. Rev. Microbiol..

[31] Brown T., Rottinghaus G., Williams M. (1992). Fumonisin mycotoxicosis in broilers: Performances and pathology. Avian Diseases.

[32] Goope N.V., Sharma R.P.H. (2003). Fumonisin B1-induced apoptosis is associated with delayed inhibition of protein kinase C, nuclear factor-kappa B and tumor necrosis factor alpha in LLC-PK1 cells. Chem-Biol. Interact..

[33] Gartner L.P., Hiatt J.L. (2007). Sistema Digestivo. Tratado de Histologia em cores.

[34] De Walle J.V., Sergent T., Piront N., Toussaint O., Schneider Y.J., Larondelle Y. (2010). Deoxynivalenol affects *in vitro* intestinal epithelial cell barrier integrity through inhibition of protein synthesis. Toxicol. Appl. Pharmacol..

[35] Marin D.E., Gouze M.E., Taranu I., Oswald I.P. (2007). Fumonisin B1 alters cell cycle progression and interleukin-2 synthesis in swine peripheral blood mononuclear cells. Mol. Nutr. Food Res..

